# Case report of the evidence of a spontaneous Reverse Pulfrich effect in monovision after cataract surgery

**DOI:** 10.1186/s12886-023-03041-w

**Published:** 2023-06-23

**Authors:** Victor Rodriguez-Lopez, Carlos Dorronsoro

**Affiliations:** 1 Institute of Optics, Spanish National Research Council (IO-CSIC), Serrano 121, Madrid, Spain; 2 2EyesVision SL, Madrid, Spain

**Keywords:** Case report, Cataracts, Monovision, Pulfrich effect, Blur, Adaptation, Depth misperception

## Abstract

**Background:**

Cataracts affect the optics of the eye in terms of absorption, blur, and scattering. When cataracts are unilateral, they cause differences between the eyes that can produce visual discomfort and harm binocular vision. These interocular differences can also induce differences in the processing speed of the eyes that may cause a spontaneous Pulfrich effect, a visual illusion provoking important depth misperceptions. Interocular differences in light level, like those present in unilateral cataracts, can cause the Classic Pulfrich effect, and interocular differences in blur, like those present in monovision, a common correction for presbyopia, can cause the Reverse Pulfrich effect. The visual system may be able to adapt, or not, to the new optical condition, depending on the degree of the cataract and the magnitude of the monovision correction.

**Case presentation:**

Here, we report a unique case of a 45-year-old patient that underwent unilateral cataract surgery resulting in a monovision correction of 2.5 diopters (D): left eye emmetropic after the surgery compensated with a monofocal intraocular lens and right eye myopic with a spherical equivalent of -2.50 D. This patient suffered severe symptoms in binocular vision, which can be explained by a spontaneous Pulfrich effect (a delay measured of 4.82 ms, that could be eliminated with a 0.19 optical density filter). After removing the monovision with clear lens extraction in the second eye, symptoms disappeared. We demonstrate that, at least in this patient, both Classic and Reverse Pulfrich effects coexist after unilateral cataract surgery and that can be readapted by reverting the interocular differences. Besides, we report that the adaptation/readaptation process to the Reverse Pulfrich effect happens in a timeframe of weeks, as opposed to the Classic Pulfrich effect, known to have timeframes of days. Additionally, we used the illusion measured in the laboratory to quantify the relevance of the spontaneous Pulfrich effect in different visual scenarios and tasks, using geometrical models and optic flow algorithms.

**Conclusions:**

Measuring the different versions of the Pulfrich effect might help to understand the visual discomfort reported by many patients after cataract surgery or with monovision and could guide compensation or intervention strategies.

## Background

The Pulfrich effect is a subtle binocular vision alteration that appears with small amounts of interocular differences in the image of the eyes [1] and can destabilize binocular vision [2–5], introducing important distortions in the perception of depth of moving objects. In its classical description, with an imbalance in retinal illuminance between eyes, the signal of the dimmer eye is processed slower than the signal of the brighter eye. It was recently discovered that interocular differences in retinal blur can also produce a misperception of depth of moving objects, the Reverse Pulfrich effect [6, 7]. The blurrier image of one eye is processed faster than the sharper image of the other eye, producing depth misperceptions of the opposite sign to the Classic Pulfrich effect.

The physical sources of the Pulfrich effect (i.e., interocular retinal illuminance or blur) can appear or change several times throughout life, increasing or decreasing as the eyes change their refraction or prescription, age, or undergo surgeries. Neural adaptation constantly adjust the visual system to the new signal, in timeframes that expand from fractions of seconds to months [8–12]. Some diseases such as optic neuritis, anisocoria, retinal diseases, and cataracts provoke neural processing delays and are sources of spontaneous Classic Pulfrich effect [13–18], but spontaneous Reverse Pulfrich has not been described.

There might be clinical situations where both versions of the Pulfrich effect coexist. The natural aging of the eye produces i) presbyopia (the loss of the ability to focus near objects, affecting 100% of the population over 45 years old) changing the refractive state of the eye and introducing important amounts of blur in near vision, and ii) reduces the transmittance of the crystalline lens decreasing the total amount of light reaching the retina compared to a young eye [19]. Along the same lines, cataract is a pathological condition also related to aging that produces a further reduction in transmittance due to the oxidation of the proteins of the tissue of the crystalline lens [20], and thus, in some scenarios, cataracts can be approximated as a neutral density filter that blocks part of the incoming light. Previous studies have reported symptoms of unilateral cataracts directly related to the Classic Pulfrich effect (differences in light between the eyes) such as difficulties during driving, walking, or in daily activities such as pouring out liquids or placing the key into the lock [15–17].

Besides, cataract is not a sudden effect. Its progressive growth allows the visual system to adapt to the interocular differences in retinal illuminance and therefore might reduce or eliminate the spontaneous Classic Pulfrich effect [21]. It has been shown, using neutral density filters, that the visual system can adapt gradually to high differences in retinal illuminance between the eyes [22–25], and that the readaptation to the initial condition is remarkably quick, in terms of days [22]. But the clinical situation is not that simple. Cataract often entails a slow reduction of contrast (due to scattering) and a progressive introduction of blur, contrast provoking the Classic Pulfrich effect, and the blur the Reverse Pulfrich effect, of opposite signs.

In cataract surgery, the crystalline lens is removed and replaced by a transparent intraocular lens (IOL), which usually compensates for the refractive error and restores the transmittance (the one associated with both the cataract and the natural aging process), all at once. The usual surgical procedure operates one eye first to avoid potential problems affecting both eyes, but the timing for the second surgery is not well established [17]. This approach likely produces interocular imbalances in scattering, transmittance (transparent intraocular lens vs. aged -or even cataractous- crystalline lens), and optical power (corrected eye vs. natural refractive error, producing not only blur differences but also magnification differences).

If the difference in retinal illuminance, contrast, or blur suddenly disappeared (for example after bilateral surgery), initial adaptation fails. Previous adaptation induces an opposite-delayed response, potentially causing sudden strong symptoms [16] until a new readaptation period, with a similar timeframe, compensates for them again. The abrupt changes after cataract surgery are particularly important with surgically induced monovision, that besides removing scattering and absorption in at least one eye (unbalancing the adaptation to the Classic Pulfrich effect), induces interocular blur (provoking the Reverse Pulfrich effect). Evidence of neural adaptation to the Reverse Pulfrich effect after surgically inducing monovision, or readaptation after removing it, has not been previously reported.

We describe a unique case of a real patient corrected with surgical monovision of 2.50 D after unilateral cataract surgery, who reports important symptoms related to spontaneous Pulfrich effect, and we analyze the conjunction between Classic and Reverse Pulfrich effects. The evolution of the patient in terms of spontaneous Pulfrich, Classic Pulfrich, and Reverse Pulfrich effect sizes, and depth misperceptions, was monitored for several months. To understand the impact in the real world of the depth misperceptions produced by the evolving Pulfrich effects described here, we also provide an estimation of the illusion size after adaptation and readaptation, by modeling the effects by combining basic geometry and optic flow algorithms in a walking environment with different types of terrains. In this representative case, the current methodology demonstrated to be useful in i) finding the origin of this spontaneous Pulfrich effect, ii) monitoring the progression of the spontaneous Pulfrich effect, iii) demonstrating the neural adaptation to the Reverse Pulfrich effect and estimating its time frame, and iv) showing the impact of spontaneous Pulfrich effect due to monovision in daily visual tasks.

## Case presentation

### Case report

A 45-year-old male with mild myopia in both eyes attended the eyecare clinic (Fundacion Jimenez Diaz, Madrid, Spain) reporting blurry vision and glare in one eye. During that visit, he was diagnosed with a subcapsular cataract in the left eye (LE), which reduced Visual Acuity (VA) to 0.4 logMAR with his best correction. The right eye (RE) was considered transparent, with normal VA (0 logMAR).

One month later, the crystalline lens of the LE was surgically removed and substituted by a monofocal intraocular lens (IOL) which compensated for the refractive error of that eye, leaving a spherical equivalent of 0.00 D. Combined with the refractive error of the RE (-2.50 D spherical equivalent), this first surgical procedure resulted in an effective monovision of 2.50 D.

In the first revision visit, one month after surgery, the patient reported difficulties in binocular vision. Distances in depth were hard to estimate when walking downstairs (and, to a minor extent, upstairs). While walking in corridors, the observation of the floor and walls produced visual discomfort. The patient, an amateur mountain runner and cyclist, refrained from practicing these activities due to insecurities and a lack of visual control of actions. Monocular VA was good (0 logMAR) in both eyes. The symptoms experienced by the patient in binocular vision could not be explained by conventional eye tests carried out in the clinic.

Symptoms did not disappear even 5 months after surgery, and the patient reported binocular vision problems and severe discomfort during this period. Six months after that first surgery in the LE, the patient underwent clear lens extraction surgery at the RE. An IOL was implanted to also correct the RE for far vision, reversing the previous monovision. After recovering from the second surgery, the patient reported that the discomfort had been alleviated and all the visual symptoms had disappeared.

In between surgeries, we started an independent non-interventional longitudinal monitoring of the patient, focused on binocular vision, at our research laboratory (Visual Optics and Biophotonics Lab, Institute of Optics, Madrid, Spain), where the patient, a scientist himself, had indirect research connections. From now on, we will refer to ‘the patient’ (of the clinic) as ‘the subject’ (of the case report). Using conventional psychophysical techniques (described in the *Methods* section), we measured the spontaneous Pulfrich effect of the subject, the Classic Pulfrich effect size, and the Reverse Pulfrich effect size, 4 weeks before the second surgery and 3 times after the second surgery (in weeks 3, 11, and 26). Each measurement took about 1 h to be performed. The results of the measurements were not shared with the patient/subject, nor with the clinic.

### Measurements

Table 1 summarizes the longitudinal evolution of the patient across time (weeks -4 to 26, being week 0 the time point of the surgery in the second eye). Symptoms were very relevant before the second surgery (measurement A; with an IOL in the left eye but not in the right eye), but not later (measurements B, C, and D; with IOLs in both eyes). The table shows, for each time point and each eye, the refractive error, and the visual acuity. The table also summarizes the main results of this longitudinal study: the quantitative estimation of the spontaneous, Classic, and Reverse Pulfrich effects, in terms of delays (interocular processing speed differences). To measure the spontaneous Pulfrich effect, the spontaneous delay in milliseconds (ms) was measured with no alteration in either eye. The Classic Pulfrich effect in milliseconds/optical density (ms/OD) was measured with neutral density filters in one eye and then in the other eye. And the Reverse Pulfrich effect in milliseconds/diopter (ms/D) was measured with optical defocus induced with trial lenses in one eye and then in the other eye.

**Table 1:** Temporal evolution of the symptoms, optical condition, refraction, and visual acuity (VA), and delay from spontaneous Pulfrich, Classic Pulfrich effect size, and Reverse Pulfrich effect size. Between weeks -4 and 3, the subject suffered cataract surgery in the right eye (RE), reversing the monovision correction and matching interocular luminance differences. IOL means intraocular lens. Asterisk (*) in the refraction means that the subject did not have the refraction corrected (therefore inducing a monovision correction)

Measurement			A	B	C	D
Week			-4	3	11	26
Symptoms			Severe symptoms. Difficulty while walking and practicing sports (trekking, running, cycling) due to distortions in ground, floor, stairs, walls. Only visually comfortable while not moving	No symptoms	No symptoms	No symptoms
Optical Condition	LE	IOL		IOL	IOL	IOL
	RE	50-year-old crystalline lens		IOL	IOL	IOL
Refraction (D)	LE	0.00		0.00	0.00	0.00
	RE	-1.75–1.50 × 160º*		-1.50 × 150º	-1.50 × 150º	-1.50 × 150º
Spherical Equivalent (D)	LE	0		0	0	0
	RE	-2.50*		-0.75	-0.75	-0.75
VA (logMAR)	LE	0.0		0.0	0.0	0.0
	RE	0.0		0.0	0.0	0.0
Spontaneous Pulfrich (delay in ms)			-4.82	-1.90	-0.54	-0.78
Classic Pulfrich (ms/OD)			-24.39	-17.81	-16.96	-13.63
Reverse Pulfrich (ms/D)			-0.03	0.62	0.64	0.27

**Fig. 1:**
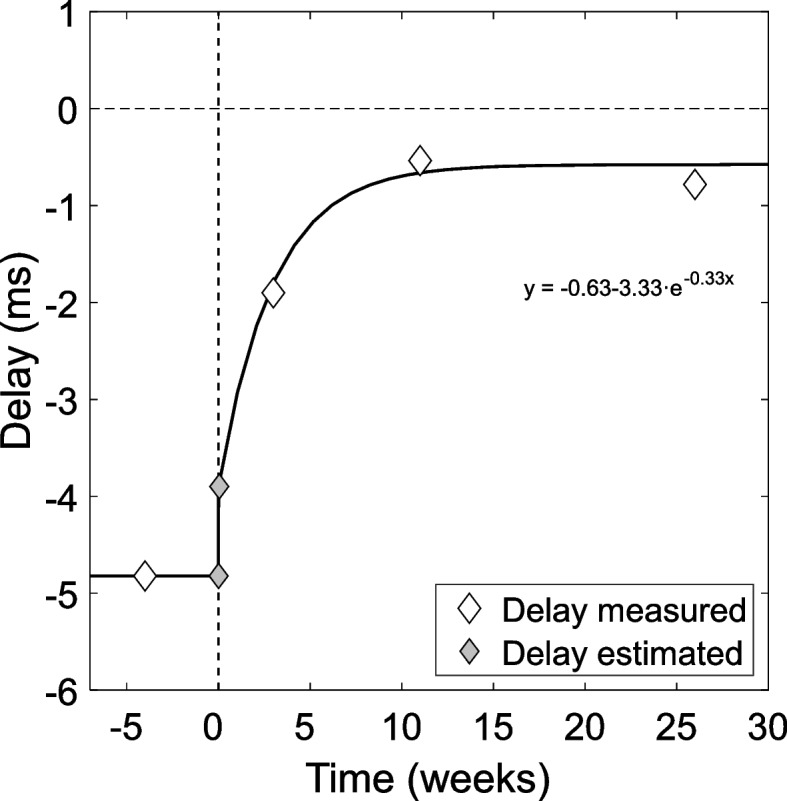
Changes in spontaneous Pulfrich effect across time. Change in neural delay, in milliseconds, across time, in weeks. Positive values indicate that the left-eye image processing speed is delayed with respect to the right-eye image, and negative values that the right-eye image processing speed is delayed with respect to the left-eye image. White diamonds indicate actual measurements (A, B, C, and D) and gray diamonds indicate estimations (A’ and A’’). The process of readaptation to the Pulfrich effect after the surgery (dashed vertical line) is very clear and is mathematically described by the equation, where *x* is time in weeks and *y* is delay in milliseconds

Figure 1 shows the temporal evolution of the spontaneous delay (spontaneous Pulfrich effect) for the subject of the case. The vertical dotted line indicates the timepoint of the second surgical procedure (clear lens extraction). The negative values of the delay indicate that the processing speed of the right eye is lower (the right eye is delayed) with respect to the left eye.

#### Measurement A

The first measurement was performed five months after the first surgery (cataract surgery in the LE, see *Case report*) and 4 weeks before the second surgery (clear lens extraction in the RE). The main changes produced by the surgery are: 1) at far distances, RE was defocused and LE focused, due to monovision, potentially causing a Reverse Pulfrich effect (increment in the RE processing speed); 2) the transmission was different between the transparent intraocular lens in the LE and the aged crystalline lens in the RE, what can potentially cause a Classic Pulfrich effect (reduction in the RE processing speed). The measurements show a remarkable spontaneous Pulfrich effect of -4.82 ms, higher than any pathological Pulfrich effect previously reported [15, 26], which could explain the severe binocular vision problems after the surgery reported in the clinical history.

#### Measurement B

One month after the first measurement, the patient underwent a second eye surgery (clear lens extraction in the RE; dashed vertical line at week 0 in Fig. 1), which removed at once the monovision correction, the interocular illuminance difference, and the potential scattering. After this surgery, both eyes had the same ocular transmittance and were focused at far, and therefore no physical source for Classic nor Reverse Pulfrich effect was expected. But previous adaptation could be likely playing a role. After the second surgery, the patient reported an immediate alleviation of the binocular vision problems. However, measurement B, performed 3 weeks after the second surgery still reported a significant spontaneous Pulfrich effect of -1.90 ms. This situation, where a non-symptomatologic patient showed a significant spontaneous Pulfrich effect, has already been described [16].

#### Measurement C

Eleven weeks after the second surgery, the spontaneous Pulfrich effect was -0.54 ms, confirming the important reduction of the spontaneous delay with time.

#### Measurement D

The last measurement was performed 26 weeks after the second surgery. The value obtained (-0.78 ms) confirmed the low value of the spontaneous Pulfrich effect and the stabilization of the delay.

### Fitting and estimations

Figure 1, summarizing all the measurements, suggests a progressive readaptation of the spontaneous Pulfrich effect after the second surgery, with timeframes of months. To estimate the time constant of the readaptation decay, we fitted the measurements to an exponential function, using mean least squares. The fitting was performed with the three measurements taken after week 0 (Measurements B, C, and D) when the optical conditions were similar between eyes and across measurements. An additional data point A’’ was included in the fitting. A’’ is not a measurement but an estimation of the postoperative delay, obtained from measurement A, as described next.

It can be considered that after 5 months of evolution from the first surgery, the neural delay was stable during the four last weeks before the second surgery. Thus, point A’ in Fig. 1 (week 0, but just before the second surgery) is obtained from a flat extrapolation of the measurement performed in week -4, and represents the preoperative delay. To estimate point A’’, we assume two quick changes taking place during and right after the surgery: an immediate change in interocular transmittance during the surgery and a quick readaptation of the Classic Pulfrich effect. Artigas et al. [19] reported 96% transmittance for an intraocular lens in-eye, and 88% for a 50-year-old natural crystalline lens, the optical condition of the subject of this case. We estimated a preoperative interocular optical density difference of 0.04 OD. From that, we estimated a delay of -0.92 ms for this subject (negative because the Classic Pulfrich effect produced a delay in the RE) considering the preoperative transmittance differences and the Classic Pulfrich effect size measured (i.e., the amount of neural delay induced by a reduction in retinal illuminance in one eye; see Table 1), -24.39 ms/OD. After the clear lens extraction, transmittances become essentially equal in both eyes and in the following measurements. It is known that readaptation to interocular differences in light level happens in only a few days [22]. Therefore, we can obtain the postoperative point A’’ in Fig. 1, as the difference between point A’ (-4.82 ms) and the estimated delay caused by the preoperative interocular transmittance differences (-0.92 ms), resulting in -3.89 ms. We assume two phases in the readaptation curve: i) the quick readaptation of the Classic Pulfrich effect from A' to A’’; and ii) the long remaining readaptation period from A’’ to D (Fig. 1) corresponding to the Reverse Pulfrich effect alone. The exponential fitting to this second phase results in the following delay readaptation equation: $delay=-0.63-3.33\cdot {e}^{-0.33t}$ where *t* is time, in weeks.

### Evidence of readaptation and previous adaptation

Figure 1 shows a readaptation process after the second surgery because there is a very systematic evolution (reduction) of the delay once the interocular differences, both in luminance and blur, were eliminated. The mere existence of readaptation also implies a previous adaptation process, that in our subject took place after the first surgery (in between surgeries).

This adaptation/readaptation process could affect Classic Pulfrich, Reverse Pulfrich, or both. As already mentioned, the adaptation/readaptation to the Classic Pulfrich effect has been reported to occur in just a few days. In our case, it can only explain a minor part of the delay readaptation, and only in the first temporal step. The additional readaptation process found in our results, after eliminating the interocular blur and taking several weeks, points at a process of adaptation/readaptation to reverse the Pulfrich effect alone, not described before.

### Classic and Reverse Pulfrich effect sizes

**Fig. 2:**
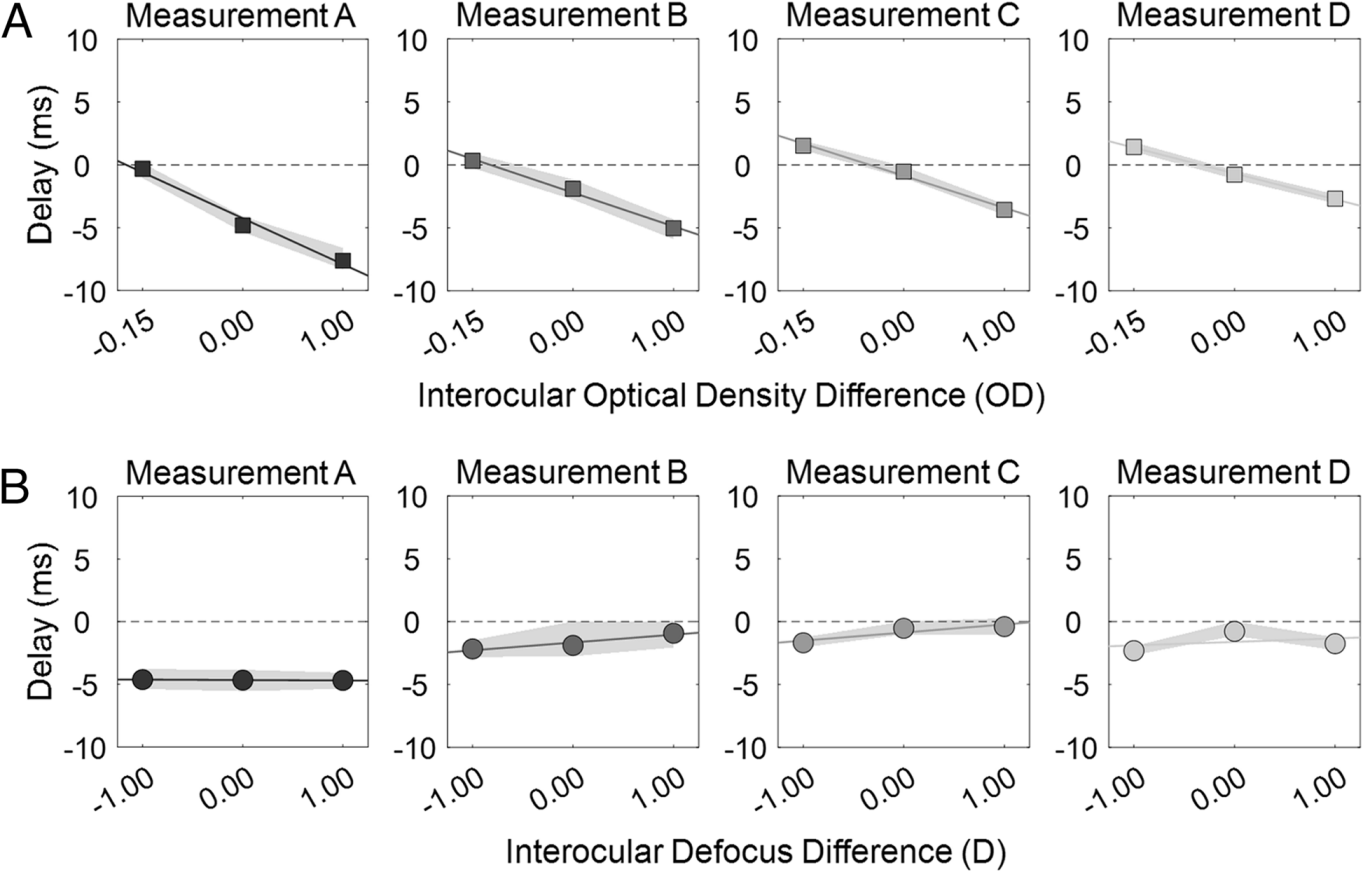
Changes in Classic and Reverse Pulfrich effects across time. **A** Classic Pulfrich. Neural delay as a function of interocular optical density difference for each measurement (A to D). **B** Reverse Pulfrich. Neural delay as a function of interocular defocus difference, in diopters, for each measurement

Figure 2 shows the temporal evolution of the Classic and Reverse Pulfrich effect sizes. Before the clear lens extraction (the second surgery), the visual system is sensitive to changes in interocular luminance, resulting (Measurement A; week -4) in a Classic Pulfrich effect size of -24.39 ms/OD, equivalent to a neutral density filter of 0.19 OD in the right eye (or transmittance 63%), consistent with the range on values previously reported in the literature [6, 7]. On the other side, the visual system, chronically exposed to 2.50 D of monovision at this time point, seems to be insensitive to interocular changes in blur, and the Reverse Pulfrich effect size measured was negligible, -0.03 ms/D.

After the clear lens extraction, in Measurement B, the Classic Pulfrich effect size was reduced (-17.81 ms/OD) and the Reverse Pulfrich effect size was increased (0.62 ms/D; comparable to other studies [7]). Classic and Reverse Pulfrich effect sizes remained quite constant with time afterward, with only a small reduction after the 23 weeks of evolution between Measurement B and D (from -17.81 to -13.63 in Classic; from 0.62 to 0.27 in Reverse). The Classic Pulfrich and the Reverse Pulfrich effect sizes measured have the expected sign, negative for the Classic Pulfrich (delaying the eye which less luminance) and positive for the Reverse Pulfrich (advancing the eye with more blur).

### Illusion size estimation

**Fig. 3:**
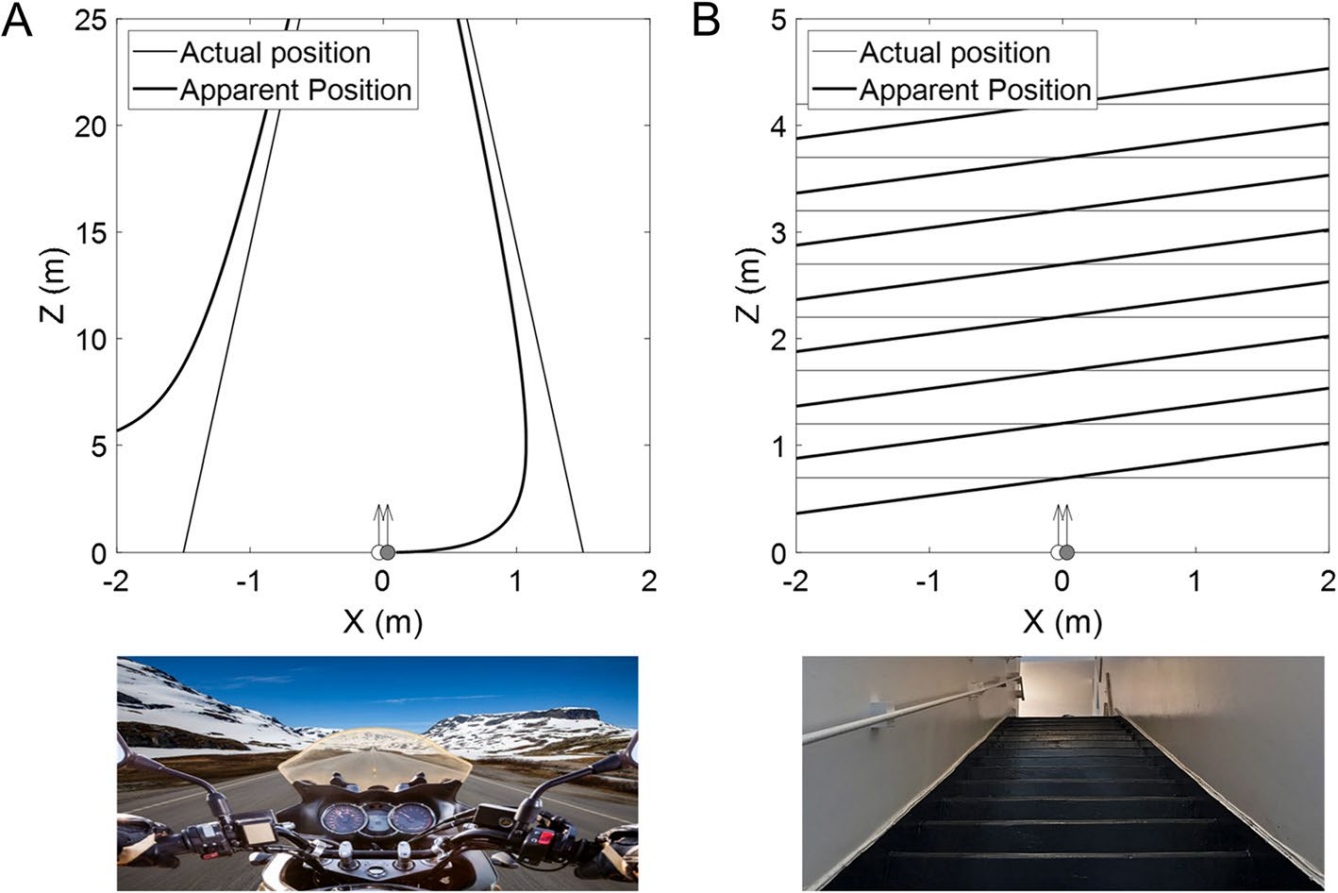
Depth misperceptions estimated for daily scenarios. Lower plots show scenes of representative situations with moving visual objects. Upper plots show the perceived trajectory of visual objects in the scenes. The observer's eyes are represented by two circles. In this case, the left eye (LE; white circles) is unperturbed, and the right eye (RE; gray circle) is delayed. The thin lines represent the actual trajectory/position of the object, and the thick lines represent the apparent trajectory/position of the object, estimated using Eq. 3 and the magnitude of the spontaneous Pulfrich effect in Measurement A (before the second surgery) when the patient presented serious symptoms (4.82 ms of delay in the RE). **A **Motorcyclist on the road. Speed considered was 40 km/h. **B **Walking down the stairs. Speed considered was 4 km/h

The illusion size in daily visual tasks was estimated for the spontaneous Pulfrich effect size measured when the patient suffered from symptoms (delay of 4.82 ms in the RE). We used the mathematical description by Spiegler [27] to geometrically estimate the disparity caused by an interocular delay and the subsequent depth misperception (*Methods* Section). In Fig. 3, we show these estimations for two situations. Thin lines represent the actual position of the object and thick lines the illusory perception. Figure 3A shows a representative example of the relative movement of the observer between two lines, like in a road lane while driving. We simulate a condition of a motorcyclist on the road, and how the lines of the road are distorted and curved due to the measured interocular delay. At 40 km/h (a common speed limit in urban areas), the depth distortion of the line could be as high as 2 m, larger than the distance from the observer to the line (1.5 m). In Fig. 3B, we simulated another condition in which the patient reported unbearable visual discomfort: walking downstairs. The simulation shows that each stair is perceived as asymmetrically deformed, complicating the simple task of setting foot on the ground. At walking speed (4 km/h), the illusion size (distortion in step height) was as high as 0.2 m.

**Fig. 4:**
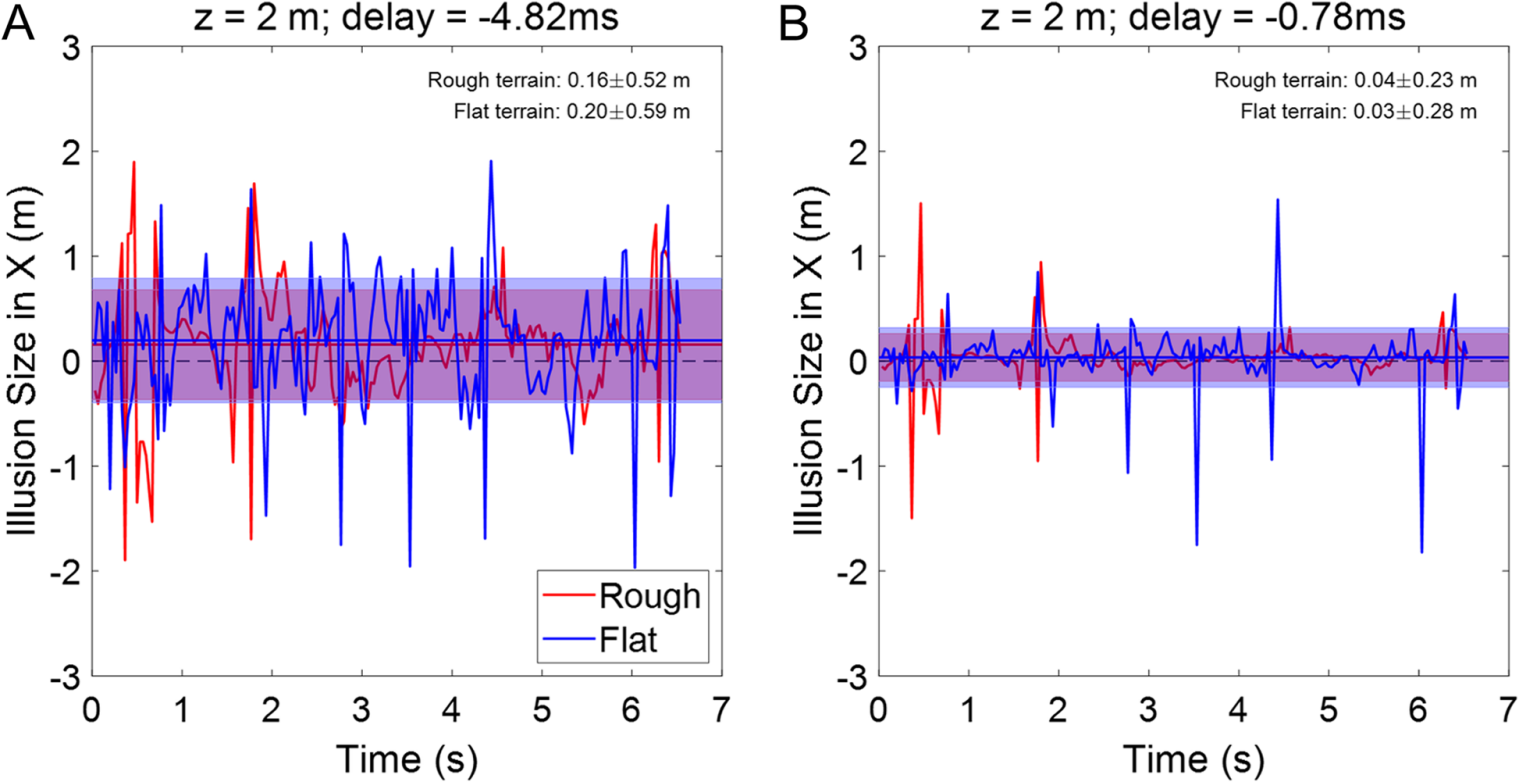
Depth misperception estimations using optic flow algorithms for different types of terrains. Illusion size in horizontal direction in meters as a function of time for an object located at 2 m distance. The shaded regions display the standard deviation in illusion size, in meters, at both sides of the average illusion size (numerical values in the upper right corner of each graph). In red, rough terrain and in blue, flat terrain. **A** Estimation of the illusion size for a delay in the processing speed of the right eye (RE) of 4.82 ms, when the patient suffered from symptoms. **B** Estimation of the illusion size for a delay in the processing speed of the right eye (RE) of 0.78 ms, when the patient did not suffer from symptoms

**Fig. 5:**
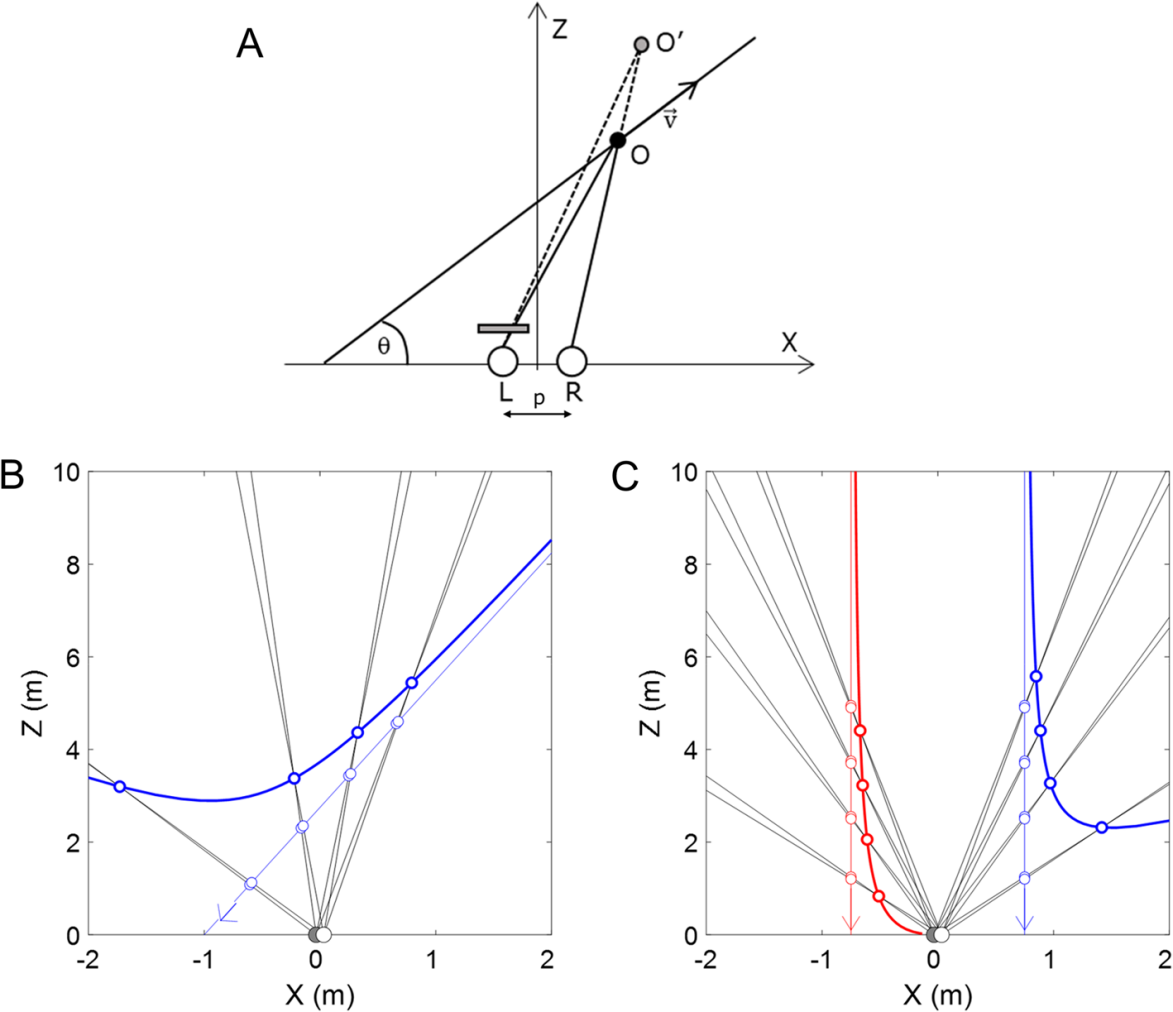
Estimating depth misperceptions caused by the Pulfrich effect using geometry. **A** Schematic diagram on the depth misperception caused by the Pulfrich effect for a given point (O) of the trajectory of a moving object at a constant speed ($\overrightarrow{v}$). The trajectory subtends an angle θ with the horizontal axis. The two white circles represent the left (L) and the right (R) eyes, and $p$ the distance between them (i.e., the interpupillary distance). A neutral density filter covering the left eye delays the processing speed of that eye with respect to the right eye, producing the object to be perceived further than its real position (O’). Adapted from Spiegler [27]. **B** Full depth misperception trajectory for the moving object represented in A, with the same neutral density filter. The object is moving at a speed of 36 km/h and at an angle θ of 70º, for an interpupillary distance of 65 mm. The left eye processing speed is delayed 5 ms with respect to the right eye. The thin blue line represents the actual trajectory of the object, and the bolded blue line the apparent trajectory due to the Pulfrich effect. Black lines represent the gaze direction of both eyes. The figure also shows four representative points along the trajectory. The object is perceived further than its real position and the magnitude of the illusion changes with the movement of the object. **C** Full depth misperception trajectory for two objects moving towards the observer in parallel trajectories separated 1.5 m (0.75 m at each side). The speed and delay are 36 km/h and 5 ms, respectively. The object to the left of the observer (thin red line) is perceived closer than its real position and finally collides with the observer (bold red line). The object to the right of the observer (thin blue line) is perceived further than its real position and moves away (bold blue line). This example can represent the lines of a lane in a road or the two walls of a corridor, each one suffering an illusion of different sign and magnitude

These two synthetic examples illustrate the importance of the effect measured, in schematic visual scenarios. However, the free observation of the real world is much more complicated. Real visual scenes contain thousands of different visual objects moving at different speeds in any direction. Retinal optic flow measurements and algorithms can quantify these movements to provide a more reasonable description of depth misperceptions (described in the *Methods* section). Figure 4 shows the illusion size while walking in different terrains (flat and rough), estimated using Eq. 3 and the retinal optic flow measured by Mattis et al. [28] (visual degrees per second, as the body, head, and eye move relative to the terrain). The illusion size was estimated for two of the interocular delays measured in the patient (-4.82 ms and -0.78 ms corresponding to Measurements A and D, in Fig. 1) for an object in the ground located at a 2 m distance. Figure 5A shows the depth estimation with time for the delay measured in Measurement A, before the second surgery, when the patient had the strongest visual discomfort. The illusion size changes chaotically as a function of time, with an average of 0.20 m for flat terrain (0.16 m for rough terrain), larger than a conventional step, and standard deviations of 0.59 m (0.52 m for rough terrain). For the delay measured in the last visit (Measurement D), 0.78 ms in the RE, when the readaptation process is complete, the average depth illusions decrease to 0.03 ± 0.28 m for flat terrain (and to 0.04 ± 0.23 m for rough terrain). The huge illusion size present in the first visit explains the difficulties reported by the patient while walking. Similarly, after monovision removal and delay readaptation, the illusion size recovers normal values, which can explain the vanishing of the visual symptoms in the same patient.

## Discussion

We report a case of spontaneous Pulfrich effect after a cataract procedure, that is induced by the combination of a Classic Pulfrich effect (interocular light difference between intraocular lens in one eye and the aged crystalline lens of the other eye) and a Reverse Pulfrich effect (interocular blur difference caused by the post-surgical monovision correction).

### Spontaneous Pulfrich effect on cataract patients

The Pulfrich effect is a well-known phenomenon affecting the binocular vision of patients with interocular differences. Therefore, it is relevant in cataract patients, where the optics of the eye changes progressively as the cataract evolves, and abruptly during cataract surgery. Scotcher et al. [29] showed a spontaneous Pulfrich effect, needing a neutral density filter of 0.25 OD -on average- to neutralize the effect, in 12 patients after cataract surgery in one eye and before cataract surgery in the second eye. In the case of the patient of this case, the neutral density filter needed was 0.19 OD. Cetinkaya et al. [17] also reported a spontaneous Pulfrich effect in 36 patients in between cataract surgeries, needing a significantly higher filter to compensate for the spontaneous Pulfrich effect (1.2 OD on average). Diaper et al. [15] reported a spontaneous delay of 1.49 and 1.16 ms in two patients with unilateral cataracts. However, the presence of a spontaneous Pulfrich effect does not always mean the presence of associated binocular vision problems [16, 17]. But the spontaneous Pulfrich effect, and the associated symptoms, are always alleviated once the interocular differences are surgically removed, typically after cataract surgery in the second eye.

The Classic Pulfrich effect is well-known and has been extensively studied. On the contrary, the Reverse Pulfrich effect is an unknown phenomenon only recently discovered in lab studies [6, 7]. For that reason, the studies in the scientific literature assume that the Classic Pulfrich effect, due to interocular luminance differences and, to a lesser extent, scattering, is the reason behind the spontaneous Pulfrich effect in between surgeries. However, both cataracts and cataract surgery also induce blur changes, which are the source of the Reverse Pulfrich effect. Therefore, the spontaneous Pulfrich effect described in the literature -and also found in this case report- could in fact be due to a Classic Pulfrich effect, a Reverse Pulfrich effect, or a combination of both. But it is difficult to identify which of the changes taking place in the optics of the eye contribute to the delay found. This case report sheds some light on the question by measuring the timeframe of readaptation to the spontaneous Pulfrich effect.

### The Reverse Pulfrich effect as the cause of the spontaneous Pulfrich effect

The patient reported severe symptoms that conditioned his lifestyle and restricted his daily activities. The discomfort was harsh enough to explain the second surgery in the healthy eye -clear lens extraction- to remove the monovision correction. Remarkably, the patient was not significantly uncomfortable in static observation. Only the symptoms caused by movements in the visual scene were unbearable for him. There is a clear parallelism between the evolution of the symptoms and the important spontaneous Pulfrich effect measured in this case report; in particular, between the reduction of the spontaneous delay with the surgery and the alleviation of the visual symptoms. The simulations and estimations of the illusion sizes from the measured delay (Fig. 4), can explain the symptoms -or absence of symptoms- described by the patient at the different stages.

But, should the spontaneous Pulfrich effect be attributed to the Classic or the Reverse Pulfrich effect? The key to answering this question is the timeline of readaptation of the patient, of several weeks, which cannot be explained by an adaptation to the Classic Pulfrich effect. Previous studies have measured the process of adaptation to interocular differences in luminance (i.e., Classic Pulfrich effect), induced on purpose with neutral density filters [22, 23, 25]. In those studies, the adaptation/readaptation to luminance difference takes periods from hours to days, but far from 10 weeks, as Fig. 1 may suggest. The long period of readaptation is not compatible with the shorter timelines of adaptation to luminance or the Classic Pulfrich effect but is fully coherent with other studies of adaptation to blur, associated with much longer adaptation periods [9–12]. At least, in this case, the readaptation process after the surgery can be attributed to a previous adaptation to a Reverse Pulfrich effect. The important spontaneous Pulfrich effect measured in between surgeries is, for the most part, due to a Reverse Pulfrich effect caused to blur and induced by monovision.

### Clinical relevance

Although recently discovered, the Reverse Pulfrich effect has already demonstrated potential implications in the clinic, due to its strong relationship with monovision correction for presbyopia. This is the first case that evidences a pathological condition caused by the Reverse Pulfrich effect.

Aniseikonia, the interocular difference in retinal magnification, is often blamed as the origin of visual discomfort when there are interocular differences in refractive power. Monovision has the potential to produce aniseikonia and for that reason it is only prescribed with contact lenses, intraocular lenses, or refractive surgery, to avoid the more important magnification associated with the vertex distance of ophthalmic lenses. The patient of this case may have suffered also from some aniseikonia. But that source of visual discomfort would be permanent -as opposed to the Pulfrich effect that only appears with moving visual objects- and seems to be minor, as the patient reported comfortable vision of static scenes. Besides, previous studies have shown that interocular magnification differences do not induce any delay [7], and therefore do not play a role in the spontaneous Pulfrich effect.

The results of this case suggest that patients with interocular blur differences who report discomfort, particularly in dynamic visual environments, should be investigated in the search for a potential Reverse Pulfrich effect. In cases where there is a Reverse Pulfrich effect, there are other strategies to deal with the symptoms beyond reverting the interocular differences. For instance, placing a filter in one eye of a patient with a spontaneous Pulfrich effect eliminate both the effect and the symptoms [15, 16]. Similarly, Rodriguez-Lopez et al. [7] proposed anti-Pulfrich monovision corrections, where the Reverse Pulfrich effect is compensated with a Classic Pulfrich effect of the same size and opposite sign. The postoperative exploration of the Reverse Pulfrich effect could be particularly useful when monovision is surgically reversible, as in light adjustable lenses [30] or laser refractive surgery.

Measurements of the Pulfrich effect have been performed using a time-consuming psychophysical paradigm (1 h per measurement). Clinical tools that provide fast, straightforward, and reliable measurements of the spontaneous Pulfrich effect may help to understand adaptation effects (of Reverse and Classic Pulfrich effects) and their relationship to inadaptation to monovision corrections. Future work needs to address massive measurements in a higher sample size to confirm the clinical relevance of the Reverse Pulfrich effect, establish normative values and the prevalence of associated visual symptoms in patients with monovision corrections.

## Conclusions

In this paper, we present the case of a patient compensated with a monovision correction after unilateral cataract surgery, who showed an important spontaneous Pulfrich effect. By longitudinally monitoring the delay after removing the interocular differences between the eyes, we demonstrate for the first time that the spontaneous Pulfrich effect was produced by a combination of Classic and Reverse Pulfrich effects. In fact, this case report shows for the first time the existence of readaptation to the Reverse Pulfrich effect, and the measurements establish a decay time of weeks, far from the timeline associated with the Classic Pulfrich effect, known to last only hours or days. Although strictly speaking we did not measure the adaptation process to the Reverse Pulfrich effect, its existence can be inferred from the measured readaptation occurring when the physical source disappeared. The very different readaptation timelines for Classic and Pulfrich effects suggest different neural adaptation mechanisms.

The unique case shown demonstrates the importance of the Pulfrich effect in interventions inducing interocular differences in retinal illuminance and blur. Measuring the spontaneous Pulfrich effect may help to understand and manage the symptoms of some patients with visual discomfort, even without differences in retinal illuminance.

## Methods

### Setup

The subject viewed the stimulus from 2 m, through well-centered trial lenses, and with his head stabilized by a chin rest with forehead support. The stimulus was shown on a 3D UK UHD 49″ monitor (LG49UH850V, LG) with a refresh rate of 60 Hz. The 3D monitor uses row-by-row spatial interlacing (i.e., the right eye sees pixels from odd rows and the left eye sees pixels from even rows) to present different images, coincident in time, to the left and the right eyes. The appropriate image for each eye was selected using passive circular polarization glasses. The spatial resolution of the display was 3840 × 2160 pixels. Only 3840 × 1080 pixels reached each eye after filtering by the polarization glasses. The maximum effective luminance of the monitor was 100 cd/m^2^.

As in previous studies of the Reverse Pulfrich effect [6, 7], the stimulus was a white moving bar of 0.125 × 1º size on a gray background. A window of 1/f noise was used to aid fusion. The movement of the bar was horizontal in space with an amplitude of 2.5º, and sinusoidal in time, with 2.5º/s of peak speed in the center of the display and 0º/s in the lateral limits (at 2.5º to the left and the right). To induce disparity in the image, we introduced subtle manipulations of the horizontal position of the moving bar for one eye (OS), following a constant interocular temporal shift ($\Delta t$, also called delay or advance).

Positive values of $\Delta t$ indicate that the RE is delayed relative to the LE, and negative values that the RE is advanced relative to the LE. When the temporal shift is zero, the bar moves on the plane of the screen. But delays and advances are translated into crossed disparities and uncrossed disparities, resulting in depth illusions that depend on the direction and speed of movement.

The task was a two-alternative forced choice (2AFC). The patient had to indicate the sign of the depth illusion (closer or further) when the bar was moving to the right or the left, obtaining a nine-level (i.e., nine temporal shifts) psychometric function. The 50% point of the psychometric means the Point of Subjective Equality (PSE) and indicated the interocular delay induced onscreen needed to null the neural delay caused by the interocular differences between the images. A more detailed explanation can be found in Burge et al. [6]. The performance of the subject of this case report was similar to that of other subjects in other studies [6, 7].

### Experiments

The subject performed the measurements with his refractive error corrected, using his usual compensation. Besides, we added an extra + 0.50 D using trial lenses to focus the screen.

To induce the Classic Pulfrich effect, we introduced “virtual” differences in light by digitally reducing the luminance onscreen by a factor equivalent to a neutral density filter with a particular optical density (OD). We produced interocular optical density differences reducing the incoming light of one eye while keeping the other one unperturbed, and vice versa. We estimated the interocular luminance difference ($\Delta O$) as the optical density difference between RE (${O}_{R}$) and LE (${O}_{L}$), as shown in Eq. 1.

1 $$\Delta O={O}_{R}-{O}_{L}$$

Similarly, to induce the Reverse Pulfrich effect, we produced interocular differences in defocus introducing defocus in one eye and keeping the other one unperturbed and vice versa. The defocus induced was always myopic defocus using concave (positive) trial lenses. The resultant retinal blur cannot be compensated by accommodation. We estimated the interocular defocus difference ($\Delta F$) as the difference in optical power between the RE (${F}_{R}$) and the LE (${F}_{L}$).

2 $$\Delta F={F}_{R}-{F}_{L}$$

negative values of $\Delta O$ and $\Delta F$ mean that the LE was perturbed and positive values that the RE was perturbed.

We measured the difference in processing speed for two conditions of $\Delta O$, ± 0.15 OD (Classic Pulfrich effect), and two conditions of $\Delta F$, ± 1.00 D (Reverse Pulfrich effect). Besides, the spontaneous Pulfrich effect was measured with both eyes equally illuminated and sharp.

The three measurements of delay for the Classic Pulfrich effect (negative optical density, no optical density, and positive optical density) were linearly fitted via a least-squares regression. The slope (delay vs optical density) indicates the Classic Pulfrich effect size. Similarly, the delays (or interocular differences in processing speed) for the Reverse Pulfrich effect (negative defocus difference, no defocus difference, and positive defocus difference) were linearly fitted to provide the size of the Reverse Pulfrich effect.

To evaluate the evolution with time before and after the second surgery, we considered those three metrics: 1) the spontaneous Pulfrich effect, i.e., the direct measurement of the neural delay without any induced disturbances in any eye; 2) the Classic Pulfrich effect size; and 3) the Reverse Pulfrich effect size.

### Estimating depth misperceptions

The delays (or interocular differences in processing speed) caused by the different versions of the Pulfrich effect and measured according to the procedures described in the previous section, can produce misperceptions in depth. The magnitude of these misperceptions not only depends on the interocular differences in blur or luminance, but also on other factors such as distance of observation, the direction of motion, and the speed of the target with respect to the observer, or interpupillary distance of the observer. Spiegler [27] derived from the problem geometry the equations to predict the misperceptions caused by a target moving at a constant speed (Fig. 5A). A detailed derivation of the equations can be found in the original manuscript [27].

A couple of representative examples can be found in Fig. 5B and Fig. 5C. In these examples, only the left eye was delayed (${t}_{R}=0$). The processing speed of the left eye image is delayed 5 ms with respect to the right eye image. In Fig. 5B, the apparent trajectory (thick blue line) dramatically differs from the actual trajectory (thin blue line), with differences as high as 4 m. Figure 5C shows an object approaching in a straight line of 0.75 m separated to the left (red line) and the right (blue line) of the observer. For the object approaching from the left, the object appears to move towards the face of the observer. For the object approaching from the left, its illusory trajectory escapes from the observer at about 2 m. As shown in these examples, a couple of milliseconds could represent a huge misperception in depth.

This mathematical description can estimate any misperception produced by objects moving in a predictable trajectory with respect to the eyes of the observer, assuming stable fixation without head movements: targets moving in front of the observer (e.g., a bicycle intersecting a road), targets that appear laterally from the field of view (e.g., a car in the other lane of a highway), targets moving towards the observer (e.g., a baseball ball), or static objects perceived as moving objects due to the relative motion of the observer (e.g., walls, steps, obstacles, lines, handrails, etc.). However, in the real visual world, with gaze changes and head movements, visual objects move across the visual field with unpredictable trajectories and at changing speeds, creating a complex and chaotic visual depth environment with considerable spatiotemporal variations which, in presence of a spontaneous interocular delay, are likely to produce a strong visual discomfort. The analytical description of Spiegler [27] may underestimate the potential implication of Pulfrich's misperceptions in real-world scenes. Retinal optic flow can provide a more realistic description of the motion of the objects in a scene, and therefore of the resultant depth misperceptions. Retinal optic flow refers to the apparent motion of objects in the retina caused by the relative motion of the observer (body, head, and eyes) and the visual scene.

We have used the dataset from Mattis et al. [28], describing the change in optical flow in visual degrees as a function of time for a subject walking through flat (easy to walk) and rough (rocky, more difficult to walk) terrains. For estimating the optic flow, they used eye-tracking, but they also monitored head and body movements. For the purposes of the Pulfrich effect, only the horizontal component of the retinal optic flow is relevant (the vertical component will not create a meaningful Pulfrich effect). Thus, we estimated the speed of change of optical flow *v* for every time frame. The depth misperception caused for every frame was estimated using the following equation.

3 $$\widehat{d}=\frac{p}{p+v\cdot \Delta t}\cdot d$$

where $\widehat{d}$ is the apparent position in depth, $p$ is the interpupillary distance, and $d$ is the actual position. We have computed the potential depth misperception for the first and the last measurement of the spontaneous Pulfrich effect of the patient, for an object located at 2 m.

## Data Availability

All data generated or analyzed during this case report are included in this published article.

## Abbreviations

RE: Right eye
LE: Left eye
D: Diopter
OD: Optical density
VA: Visual acuity
IOL: Intraocular lens
